# Methods for Applying Accurate Digital PCR Analysis on Low Copy DNA Samples

**DOI:** 10.1371/journal.pone.0058177

**Published:** 2013-03-05

**Authors:** Alexandra S. Whale, Simon Cowen, Carole A. Foy, Jim F. Huggett

**Affiliations:** Molecular and Cell Biology Team, LGC Ltd, Teddington, United Kingdom; Institute of Molecular Genetics IMG-CNR, Italy

## Abstract

Digital PCR (dPCR) is a highly accurate molecular approach, capable of precise measurements, offering a number of unique opportunities. However, in its current format dPCR can be limited by the amount of sample that can be analysed and consequently additional considerations such as performing multiplex reactions or pre-amplification can be considered. This study investigated the impact of duplexing and pre-amplification on dPCR analysis by using three different assays targeting a model template (a portion of the *Arabidopsis thaliana* alcohol dehydrogenase gene). We also investigated the impact of different template types (linearised plasmid clone and more complex genomic DNA) on measurement precision using dPCR. We were able to demonstrate that duplex dPCR can provide a more precise measurement than uniplex dPCR, while applying pre-amplification or varying template type can significantly decrease the precision of dPCR. Furthermore, we also demonstrate that the pre-amplification step can introduce measurement bias that is not consistent between experiments for a sample or assay and so could not be compensated for during the analysis of this data set. We also describe a model for estimating the prevalence of molecular dropout and identify this as a source of dPCR imprecision. Our data have demonstrated that the precision afforded by dPCR at low sample concentration can exceed that of the same template post pre-amplification thereby negating the need for this additional step. Our findings also highlight the technical differences between different templates types containing the same sequence that must be considered if plasmid DNA is to be used to assess or control for more complex templates like genomic DNA.

## Introduction

The high precision offered by digital PCR (dPCR) has the potential for measuring smaller fold changes than established techniques like quantitative real-time PCR (qPCR). This potentially offers a new tool for clinical measurement applicable to approaches such as assessment of DNA copy number variation (CNV) [1]. However, for maximum precision, the DNA sample must be at an optimum concentration [2] and this can be challenging due to the fact that clinical samples are frequently of limited size and concentration. Duplex PCR, where two targets are analysed per reaction, and sample pre-amplification offer methods that would allow an increase in the number of tests that can be performed on a sample, while also reducing the required sample size needed. Duplex PCR is also desirable in clinical settings as it offers cost and throughput benefits, although it is typically more challenging to design and optimise than conventional uniplex PCR. Aspects of duplex PCR that could introduce bias include a preferential amplification of one target over the other or inhibitors presence in the sample that affect one assay more than the other [3].

Pre-amplification is an increasingly used method for quantifying nucleic acid samples that have limited amounts of starting material [4], [5], contain low abundant targets of interest [6], [7] or for high-throughput methods, whereby a large number of targets are analysed in parallel [8], [9]. Several different pre-amplification approaches to amplify either sequence-specific regions or whole genomes are available employing both PCR [10], [11] and isothermal amplification based approaches [12]–[16].

The underlying assumption, when quantifying samples post pre-amplification, is that all targets of interest are pre-amplified in equal proportion to maintain their relative quantity to that present in the original sample. A PCR-based method for pre-amplification, that can multiplex up to 100 different sequence-specific target assays [17], is being increasingly used in combination with high throughput qPCR analysis [8], [9]. Using this method, the relative amount of 10 targets has been demonstrated to be maintained with an accuracy of ≥1.5-fold [6] although a small measurement bias associated with this method of pre-amplification has also been described [18]. In this study we have performed a detailed evaluation into the impact of this pre-amplification method and duplex format on dPCR accuracy, precision and bias by investigating technical reproducibility and impact of template type. We provide recommendations for using dPCR in combination with pre-amplification and duplexing that are important when using this technique in clinical analysis and in relation to the application of reference materials.

## Materials and Methods

### Template DNA

A previously constructed pSP64 poly(A) plasmid containing the *Arabidopsis thaliana* alcohol dehydrogenase (ADH) gene fragment (GenBank: M12196) [19] was linearised with *Bgl*I as described previously [18]. Complete linearisation was confirmed using the 2100 Bioanalyzer and DNA 7500 series II kit (Agilent, West Lothian, UK) according to the manufacturer’s instructions (Figure S1A). The concentration of the linearised ADH plasmid (∼4.6 kb) was estimated by Nanodrop UV spectrophotometry using A_260_ measurements (Thermo Scientific, Massachusetts, USA) and converted to copy number [20]. Linearised ADH plasmid was diluted to 1×10^8^ copies/µl in carrier (50 ng/µl sonicated salmon sperm DNA; Agilent) and stored in aliquots at −20°C to prevent freeze-thawing effects. The concentration of purchased *Arabidopsis* genomic DNA (AMS Biotechnology Ltd, Oxfordshire, UK) was estimated and converted to copy number as described above, using the *Arabidopsis* genome size as 157 Mb [21], and stored in aliquots of 0.1 µg/µl (∼6.7×10^5^ copies/µl) at 4°C as per manufacturer’s instructions. Agarose gel electrophoresis was used to confirm high quality genomic DNA with high molecular weight (Figure S1B) For all experiments, template DNA was freshly diluted volumetrically from concentrated stock in carrier to the desired concentration.

### Primers and Probes

All primers and hydrolysis probe sequences for the three Adh assays have been described and optimised previously [18] (details also available in Table S1 and Figure S2A) in accordance with the MIQE guidelines [22] (Table S2). Each Adh assay hydrolysis probe was conjugated with either a FAM or a VIC fluorophore to give a total of six Adh assays: Adhα-FAM, Adhα-VIC, Adhβ-FAM, Adhβ-VIC, Adhδ-FAM and Adhδ-VIC (Table S1). The amplification of a single PCR product for each assay was confirmed using the 2100 Bioanalyzer and DNA 1000 kit (Figure S2B).

### Pre-amplification of Template DNA

Each 10 µl multiplex pre-amplification reaction consisted of TaqMan® PreAmp Master Mix (ABI, California, USA), 0.05× pooled sequence-specific gene assay mix and 2 µl of template DNA (∼1,000 copies/reaction). Pre-amplification no template controls (NTC) were set up in parallel with carrier DNA, but without target DNA. The pooled sequence-specific gene assay mix was prepared as a 0.2× stock containing 180 nM primers (both forward and reverse) for each of the three Adh assays in 1× TE (pH 8.0) to give a final concentration of 45 nM per primer in each 10 µl reaction. Pre-amplification was performed using the GeneAmp® PCR 9700 System (ABI) with thermocycling conditions of 95°C for 10 minutes, followed by 14 cycles of 95°C for 15 seconds and 60°C for 4 minutes. All pre-amplification reactions were diluted 1∶5 with 1× TE (pH 8.0) to give a total volume of 50 µl and stored in aliquots at −20°C to prevent freeze-thawing effects.

### Real-time Quantitative PCR

For assessment of uniplex and duplex assays, 10 µl reactions, containing TaqMan® Gene Expression Master Mix (ABI), Adh-FAM assay and/or Adh-VIC assay and 2 µl template DNA were performed on a seven-point ten-fold dilution series of the linearised ADH plasmid (∼2×10^7^ to ∼2×10^1^ copies/reaction) (Figure S2C–D & S3). qPCR was performed using the Prism 7900HT Real Time PCR system (ABI). Thermocycling conditions were 95°C for 10 minutes, followed by 40 cycles of 95°C for 15 seconds and 60°C for 60 seconds. NTCs were performed using carrier containing no template DNA; in all cases no amplification occurred (Figure S2E). The SDS software v2.4 (ABI) was used to calculate the quantification cycle (Cq) value, which is defined as the number of cycles at which the fluorescence signal is significant above the threshold. This was repeated on three separate days from a freshly prepared standard curve to give three data points for each dilution and uniplex or duplex combination.

qPCR was performed as a screening tool prior to dPCR analysis to determine the optimal dilution factor for analysis of the pre-amplification reactions by subsequent digital PCR. A dilution series of the pre-amplification reaction was performed in 1× TE (pH 8.0) (1∶5 to 1∶750) (Figure S4A). 10 µl duplex reactions containing TaqMan® Gene Expression Master Mix, the respective Adh-FAM assay (α, β or δ), Adh-VIC (β or δ) assay and 2 µl of either the diluted pre-amplification reaction or non-pre-amplified template DNA (∼6,000 or ∼1,000 copies/reaction) were performed. The Adhβ assay was present in every duplex combination investigated. Thermocycling conditions and analysis were identical to those described above. Pre-amplification NTCs were analysed (1∶5 dilution with 1× TE, pH 8.0) and PCR NTCs were performed using carrier containing reaction mix and no template DNA; in all cases no amplification occurred (Figure S4B).

### Microfluidic Digital PCR

For the initial assessment of uniplex and duplex assays on the linearised ADH plasmid, 12.765 digital PCR arrays (Fluidigm, California, USA), containing 765×6 nl chambers in each of the 12 panels, were used. Reactions of 8 µl, containing TaqMan® Gene Expression Master Mix (ABI), 1× GE sample loading reagent (Fluidigm), Adh-FAM assay and/or Adh-VIC assay and 2.5 µl template DNA, were pipetted into each of the loading inlets of a 12.765 array. The BioMark IFC controller MX (Fluidigm) was used to uniformly partition the reaction from the loading inlets into the panels. dPCR was performed using the BioMark System for Genetic Analysis (Fluidigm). Thermocycling conditions were identical to those described for qPCR. The Digital PCR Analysis software (Fluidigm) was used to count the number of positive chambers (*H*) out of the total number of chambers (*C*) per panel and the Poisson distribution was used to estimate *λ*, the average number of template copies per chamber in a panel (*λ* = −ln (1−*H*/*C*)) [23]. For the most accurate measurement using this platform it is recommended that dPCR be performed with 200–700 positive chambers per panel to give the Poisson corrected ∼230 to 1900 copies/panel (0.3< *λ* <2.5) [24]. The quality threshold (QT) value was set at 0.2 for all assays and the Cq threshold was adjusted to eliminate the impact of cross-talk between the FAM and VIC filters. For each 12.765 dPCR array, template DNA (∼1,000 copies/panel to give *λ* = 1.3) was analysed in triplicate panels for a duplex assay and its two corresponding FAM and VIC assays in uniplex (nine panels in total) (Figure S2F–G). The remaining three panels were used for NTC reactions using carrier containing no template DNA for each uniplex and duplex assay; in all cases no positive chambers were observed (Figure S2H). This experiment was repeated on three separate days for each duplex assay combination. The absolute counts for each experiment are given in Table S3.

To accommodate the increased replication necessary for the assessment of either the uniplex and duplex comparison on genomic DNA or the pre-amplification experiments, the 48.770 digital PCR arrays (Fluidigm), containing 770×0.84 nl chambers in each of the 48 panels, were used; differences using this array in DNA copies per µl and associated *λ* values are outlined below. Reactions of 6 µl were established containing TaqMan® Gene Expression Master Mix, 2× GE sample loading reagent, Adh-FAM assay, Adh-VIC assay and 1.2 µl of either the optimally diluted pre-amplification reaction (∼600 copies/panel to give *λ = *0.76) or non-amplified template DNA (∼600 or ∼100 copies/panel to give *λ* = 0.76 or 0.13, respectively) (Figure S4C–E). Reactions were randomly pipetted into each of the loading inlets of a 48.770 array (Fluidigm), that were uniformly partitioned into the panels followed by dPCR and analysis as described above. Unless otherwise stated, reactions were performed on quadruplicate panels. NTCs were performed using carrier containing master mix with no template DNA; in all cases no positive chambers were observed (Figure S4F). This experiment was repeated on three separate days for each duplex assay combination. The absolute counts for each experiment are given in Table S4.

### Statistical Analysis

qPCR data obtained from the SDS software v2.4 (ABI) and Digital PCR Analysis software Version 3.0.2 (Fluidigm) were exported into Microsoft Excel spreadsheets and converted into tab-delimited text. All statistical analyses were performed using MS Excel 2007 and the R statistical programming environment (http://www.r-project.org/). All copy number variation data were expressed as ratios of the Adhβ divided by the respective other Adh assay. Figures were generated using MS Excel 2007, GraphPad Prism 5 and the R statistical programming environment.

For comparison of the uniplex and duplex reactions, qPCR experiments were initially performed; linear regression was applied to each of the three independent qPCR experiments and the correlation (R^2^) and PCR efficiency (E% = (10^(−1/slope)^ −1) ×100)) computed. The uniplex and duplex reactions were tested against each other for differences in Cq and E% using the Student’s *t*-test.

The dPCR data were analysed by comparing copy number variation for uniplex with duplex assays, and analysing the non-pre-amplified compared to pre-amplified templates. The natural logarithm of the copy number ratio between the two respective assays was used as the response variable, as it has distribution properties which are close to Normal [2]. For each experiment (panel-to-panel), the mean log ratio and its uncertainty were computed using the expression for the standard error described in a previous paper [2]. Each combination of assay and plex formats were measured between the three experiments (array-to-array), and an overall mean log ratio and standard error calculated for each combination. Standard errors were calculated using analysis of variance (ANOVA), and included contributions from both within- and between-experiment variation. The homogeneity of within-group variation across groups was tested using Levene’s test, which showed no evidence of significant measurement repeatability differences between experiments, satisfying one of the assumptions of classical ANOVA. The one sample Student’s *t*-test was then used to test the log ratios for a significant difference from zero.

Molecular dropout observed in the duplex assay data was investigated by analysing the count data within and between experiments, and were treated as multinomial (more than two possible outcomes) based on the following approach. For a given chamber in a panel, there are four possible detection outcomes, double dropout (neither assay detected), single dropout (one or the other assay detected only) and no dropout (both assays detected); with the individual count numbers for each outcome summing to the total number of chambers in the panel. A multinomial log-linear model was fitted to the data set. The multinomial distribution is a generalisation of the binomial distribution (only two outcomes), and can be viewed as an extension of the distribution of positive chambers in uniplex dPCR. The model used a maximum likelihood method to estimate a best-fit probability for each detection outcome given the -plex and assay formats, taking account of experimental sources of variation and multinomial sampling error. Full details of the model are given in the Appendix S1.

## Results

### Impact of Duplex Reactions on the Accuracy and Precision of dPCR

Analysis of more than one target per reaction (multiplexing) requires careful evaluation prior to sample analysis and can take a considerable amount of time and resources [22]. For the majority of studies, duplex reactions (two targets) are used in qPCR whereby each target is detected with a different fluorophore associated with its specific assay [25], [26]. Here we have evaluated the performance characteristics of the Adh assays in both uniplex and duplex formats with qPCR followed by investigation of the effects of the assay format on the accuracy and precision of the ratio measurement between two Adh assays using dPCR.

A standard curve of the linearised ADH plasmid was analysed by qPCR for each of the six Adh assays in uniplex (three primer and probe sets with two fluorophores: Adhα-FAM, Adhα-VIC, Adhβ-FAM, Adhβ-VIC, Adhδ-FAM and Adhδ-VIC) and for the six possible combinations of the Adh assays in duplex (Adhα-FAM:Adhβ-VIC, Adhβ-FAM:Adhα-VIC, Adhα-FAM:Adhδ-VIC, Adhδ-FAM:Adhα-VIC, Adhβ-FAM:Adhδ-VIC and Adhδ-FAM:Adhβ-VIC). The linear correlation and PCR efficiencies were calculated from each standard curve (Table S5 & Figure S3). For all six assays, the PCR efficiencies for VIC-conjugated probes were significantly higher when analysed in duplex compared with their uniplex counterparts (*p = *0.02), while the PCR efficiencies for FAM-conjugated probes were unaffected by the duplex format (*p = *0.54) (Table S5). In all cases, the PCR efficiencies were >91% and the Cq values were unaffected by using the duplex format (Table S5 & Figure S3).

**Figure 1:**
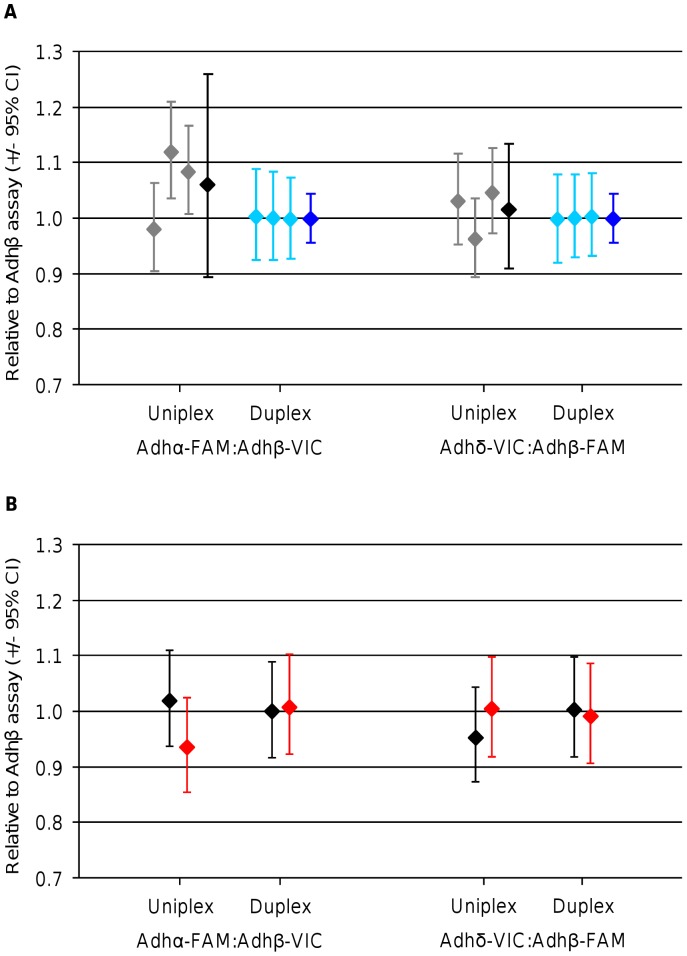
Comparison of uniplex and duplex reactions by digital PCR. (A) Graph showing the ratios calculated for the three experiments using either uniplex (grey data points) or duplex (light blue data points) reactions on the linearised ADH plasmid for two Adh ratios: Adhα-FAM:Adhβ-VIC and Adhδ-VIC:Adhβ-FAM. Each data point and its associated 95% CIs were calculated from triplicate panels on a single 12.765 dPCR array (panel-to-panel variation). The expanded uncertainty was calculated from the three experiments for each ratio using uniplex (black data points) and duplex (dark blue data points) reactions. For the uniplex reactions, the standard error of the mean for the three experiments was used to calculate the 95% CIs as the between experiment variance exceeds that of the within experiment variance. For the duplex reactions, the 95% CIs were calculated from the mean variance across the three experiments as the between and within experiment variance was very small. (B) Graph showing the ratios calculated for either linearised ADH plasmid (black diamonds) or gDNA (red diamonds) using either uniplex or duplex reactions for two Adh ratios: Adhα-FAM:Adhβ-VIC and Adhδ-VIC:Adhβ-FAM. 95% CIs were calculated from triplicate panels from a single 48.770 dPCR array. The absolute counts used to generate this figure are found in Table S3.

Two duplex assays (Adhα-FAM with Adhβ-VIC and Adhβ-FAM with Adhδ-VIC) were selected for copy number analysis using dPCR for comparison to the equivalent measurement using uniplex assays (Figure 1A). As the assays are present on the same DNA molecule and therefore linked (Figure S2A), the expected copy number ratio between each of the assays is 1. Any deviation from this ratio indicates either an inaccuracy in the measurement or some molecular fragmentation leading to separation of the assay regions. The Bioanalyzer trace showed a single 4.5 kb fragment indicating that the majority of the sample was unfragmented (Figure S1A); therefore any deviation we measured was likely to be due to technical variation. Overall analysis of the Adhα-FAM:Adhβ-VIC and Adhδ-VIC:Adhβ-FAM ratios using the 12.765 digital arrays demonstrated that the duplex assays always measured a ratio of 1.00, compared with a larger spread of ratios (between 0.96 and 1.12) for the parallel uniplex reactions across three independent dPCR experiments (Figure 1A). The within experiment (panel-to-panel) variation was similar between the uniplex and duplex assays with the between experiment (array-to-array) variation being the source of the loss of precision for the uniplex reactions (Figure 1A).

Calculating the expanded uncertainty from the three experiments demonstrated that there was no significant difference between the measured and expected ratios for either uniplex or duplex reactions for both the Adhα-FAM:Adhβ-VIC ratio (*p = *0.28 and *p* = 0.97, respectively) and Adhδ-VIC:Adhβ-FAM ratio (*p = *0.69 and *p = *0.99, respectively) (Figure 1A). To evaluate uniplex and duplex assay performance on more complex gDNA a further experiment was performed using a 48.770 digital array. Analysis of the uniplex and duplex reactions demonstrated that genomic DNA performed in a comparable way to the linearised ADH plasmid for both the Adhα-FAM:Adhβ-VIC and Adhδ-VIC:Adhβ-FAM ratio (Figure 1B). Consequently, although the ratios measured using the uniplex assays gave a ratio that varied from 1, the experiment measured no bias between the two assay formats (Figure 1).

### Evaluation of Pre-amplification on Measurement Bias

We have previously observed a two-fold quantification bias when using a sequence-specific PCR-based pre-amplification approach [18]. We also demonstrated that dPCR was capable of measuring with good precision DNA concentrations at approximately 10 copies per panel (*λ* = 0.013) across a range of template types [18]. This study raised the question whether, when faced with a limited sample, it would be better to use the lower concentration, with associated reduced sensitivity, or perform pre-amplification to increase the template concentration and thus accuracy of dPCR, but at the risk of introducing bias associated with this additional step.

**Figure 2:**
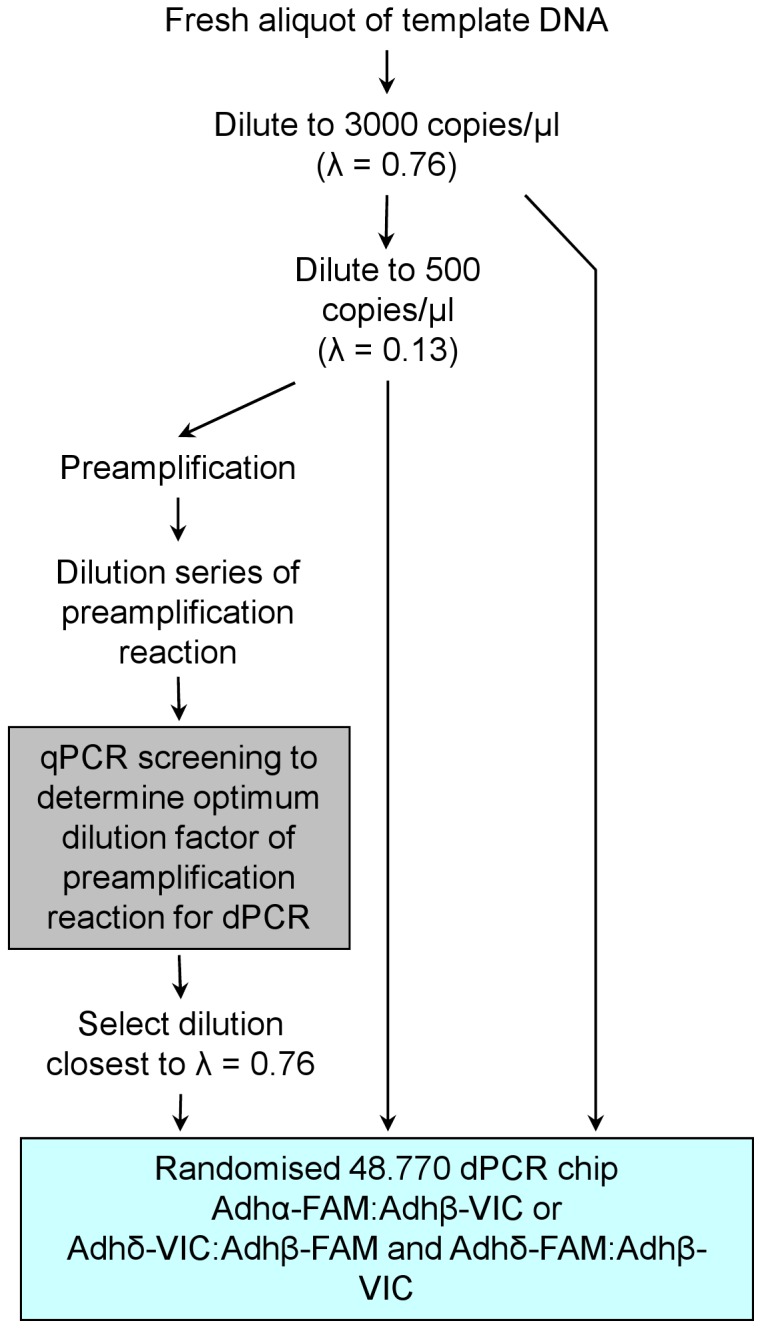
Workflow of pre-amplification experiments. A fresh aliquot of template DNA (linearised ADH plasmid or *Arabidopsis* gDNA) was diluted in carrier to a ‘high’ concentration (3000 copies/µl to give *λ* = 0.76) and a ‘low’ concentration (500 copies/µl to give *λ* = 0.13). The ‘low’ concentration was used as the template in the pre-amplification reaction. The pre-amplification reaction was serially diluted in 1× TE (pH 8.0) and using qPCR, the dilution to give an approximately *λ* = 0.76 for the Adhβ target was selected. dPCR analysis using the 48.770 arrays was performed with the Adhα-FAM:Adhβ-VIC duplex assay for the ‘high’ and ‘low’ concentration non-amplified template DNA and diluted pre-amplified template DNA. This experimental workflow was repeated on three separate days. A further three experiments were performed on three separate days using the Adhδ-FAM:Adhβ-VIC and Adhδ-VIC:Adhβ-VIC duplex assays.

**Figure 3:**
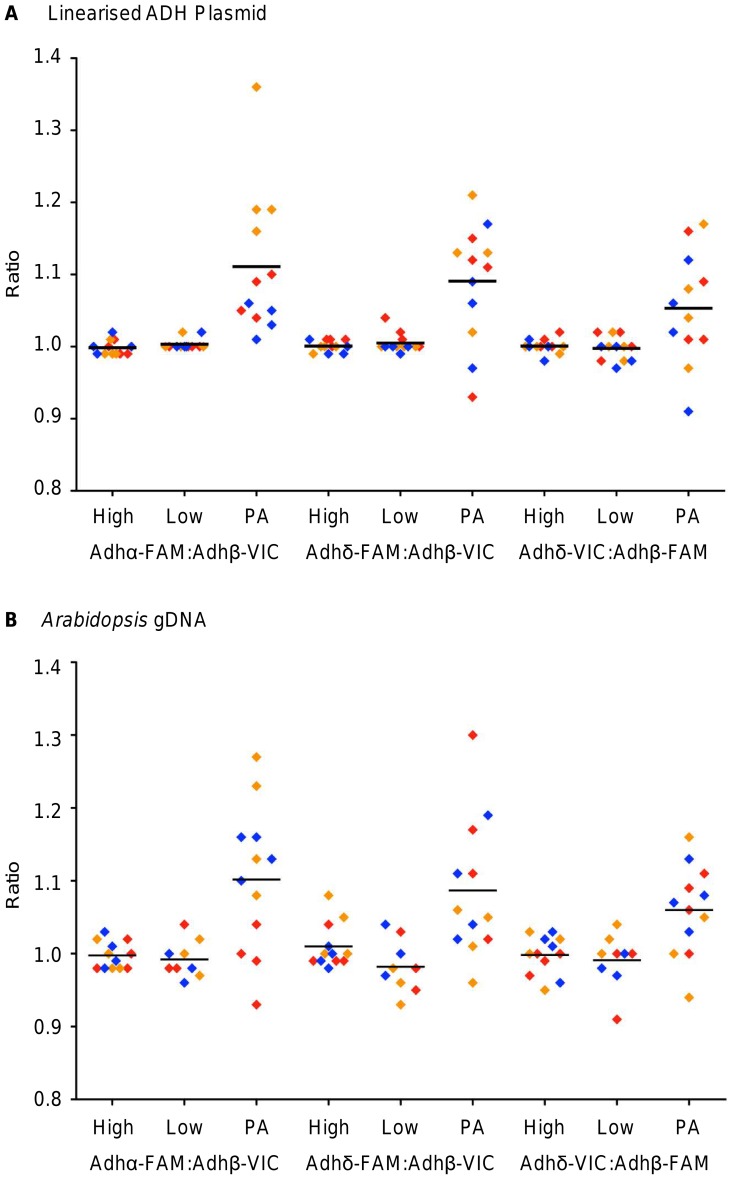
Assessment of the pre-amplification reaction on the A) linearised ADH plasmid or B) *Arabidopsis* gDNA. For each duplex assay combination, two concentrations of non pre-amplified template (High and Low) were analysed in parallel with the pre-amplified template (PA). Each sample was analysed on quadruplicate panels, with the exception of the ‘low’ non pre-amplified *Arabidopsis* gDNA that was analysed on triplicate panels, and the copy number ratio between the two Adh targets was calculated for each panel (diamond data points). Three experiments were performed (red, orange and blue diamonds) with three duplex assay combinations (Adhα-FAM:Adhβ-VIC, Adhδ-FAM:Adhβ-VIC and Adhδ-VIC:Adhβ-FAM). Horizontal black bars represent the mean ratio across all three experiments. The absolute counts used to generate this figure are found in Table S4.

Using our ‘linked molecule’ design, we have compared low concentration template (*λ* <0.13) with pre-amplified template to determine which template gave the most accurate measurement of the copy number ratio between the Adh assays (Figure 2). Two concentrations of non pre-amplified linearised ADH plasmid template DNA were analysed using the 48.770 dPCR arrays: the ‘high’ (∼3,000 copies/µl or ∼600 copies/panel to give *λ* = 0.76) that represents a measureable template that can be quantified accurately by dPCR, and the ‘low’ (∼500 copies/µl or ∼100 copies/panel to give *λ* = 0.13) that represents a template that falls below the recommended range of accurate quantification by dPCR [24]. The ‘low’ was also used as template for the pre-amplification reaction which was subsequently diluted to a concentration comparable to that of the ‘high’ for dPCR analysis (Figure 2 & Figure S4). dPCR was performed using duplex Adh assays on both the ‘high’ and ‘low’ non pre-amplified and pre-amplified DNA templates and the ratio between the two Adh assays calculated. This experiment was performed on three separate days from freshly diluted template DNA using the Adhα-FAM:Adhβ-VIC duplex assay. To investigate the impact of using different fluorophores, a further three experiments were performed using this design (Adhδ-FAM:Adhβ-VIC and Adhδ-VIC:Adhβ-VIC duplex assays). In all cases, the copy number ratio was calculated relative to the Adhβ assay (Figure 3A).

Using the Adhα-FAM:Adhβ-VIC duplex assay, analysis of the linearised ADH plasmid demonstrated that in all three experiments, the pre-amplification reaction measured a different mean Adhα:Adhβ ratio (1.07, 1.22 and 1.04). Conversely, both concentrations of the non pre-amplified templates measured an Adhα:Adhβ ratio of 1.00 for each of the three experiments (Figure 3A). A similar pattern was observed using the Adhδ-FAM:Adhβ-VIC duplex assays, where the Adhδ:Adhβ ratios for the pre-amplified template (1.08, 1.12 and 1.07) were larger than their respective non-amplified template (both concentrations measured ratios between 1.00 and 1.02) (Figure 3A). Swapping the fluorophores of the Adhβ and Adhδ assays (Adhδ-VIC:Adhβ-FAM duplex assay) did not affect the result (Figure 3A).

For all three duplex assays, the within experiment (panel-to-panel) precision was larger in the pre-amplified templates compared with their respective ‘high’ concentration non pre-amplified template. Additionally, the between experiment (array-to-array) variability of the three pre-amplification reactions was larger than that calculated for the ‘high’ concentration non pre-amplified template (Figure 3A). In all but one experiment, the within and between experiment variation of the ‘low’ concentration non-amplified template was similar to that of the ‘high’ concentration non-amplified template (Figure 3A) thereby demonstrating that although the ‘low’ concentration falls outside of the recommended range for accurate dPCR, in this experiment, it was favourable to analyse it without performing pre-amplification. These findings agree with previously reported results where dPCR has been shown to measure low nucleic acid concentrations with good precision [18].

To further assess the pre-amplification reaction using a different template, *Arabidopsis* gDNA was also analysed using the same experimental workflow (Figure 2). As observed with the linearised ADH plasmid, the ratios from the three experiments were more variable for the pre-amplified template (between 0.99 and 1.18) compared with the two concentrations of non-pre-amplified template (between 0.98 and 1.03) across the three Adh duplex assays (Figure 3B). For all three duplex assays, both the within and between experiment variability of the pre-amplification reactions exceeded that of their respective ‘low’ concentration non pre-amplified templates, which was similar to that of the ‘high’ concentration (Figure 3B).

### Impact of Template Type on dPCR Technical Variability

When performing qPCR, measurement variability occurs for two broad reasons: A) variability in the amount or quality of template added, influenced by all upstream steps required to store, extract and prepare the sample and reaction, and B) the inherent technical variability of the qPCR, expressed as the Cq readout. Digital PCR is unaffected by typical variation in the Cq, which might explain why it is more precise than qPCR [2]. However, while dPCR is also susceptible to variability in the amount of template added, technical variation also manifests as molecular dropout (where a molecule is present, but does not amplify). In this study we used a ‘linked molecule’ approach, where all three amplicons are located on the same molecule. Therefore, positive amplification of one assay demonstrates the presence of the DNA template in that dPCR chamber and therefore the other assay in the duplex reaction should also display positive amplification. We counted the number of single assay positive chambers to provide an estimate of where and how frequently molecular dropout occurred in both assays (double dropout). A multinomial modelling approach was used to analyse the data based on the possible detection outcomes: double dropout (neither assay detected), single dropout (one assay detected) and no dropout (both assays detected) (Appendix S1). This approach was particularly useful, as it modelled the events as probabilities (and hence expected numbers of chambers), taking into account the constraint imposed by the panel size (Appendix S1).

**Figure 4:**
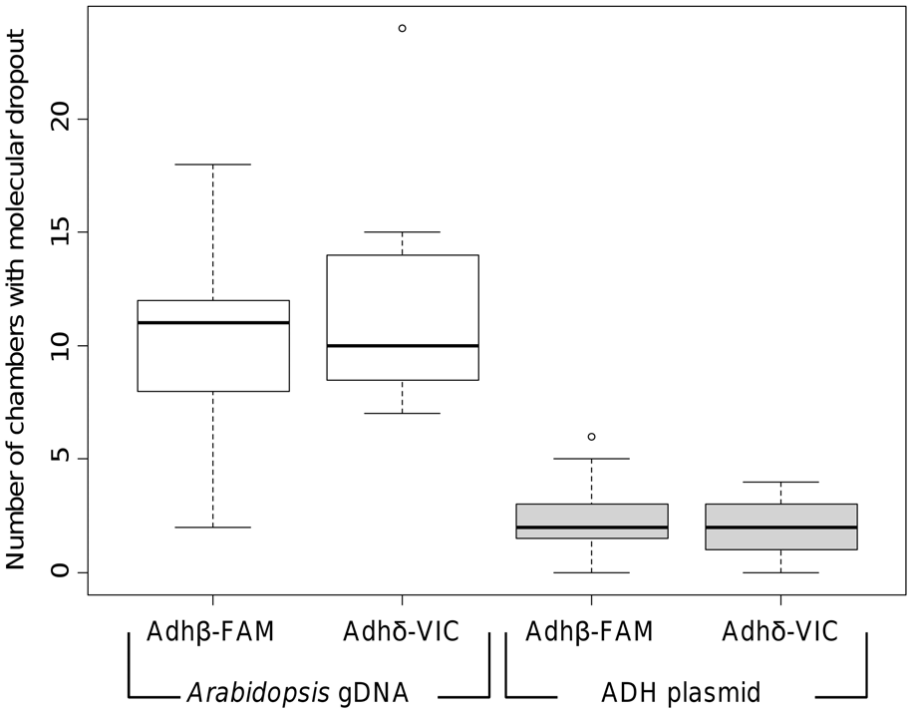
Assessment of molecular dropout by digital PCR. Box and whisker plot showing the effect of using different template types: *Arabidopsis* gDNA (white plots) or linearised ADH plasmid (grey plots) on molecular dropout in the data from the ‘high’ concentration template using the Adhδ-VIC:Adhβ-FAM duplex assay. The vertical axis corresponds to the number of chambers per panel in which known dropout occurred, that is, one assay (labelled with FAM or VIC) did not produce a positive signal, but the other assay did. For each data set the box plot represent the inter-quartile range with the mean, the whiskers represent the 95% range. The full range of the data set is represented by a circle.

No statistically significant effects associated with assay pair, fluorophore or template concentration were found. However, the occurrence of molecular dropout was significantly higher in the *Arabidopsis* gDNA when compared with the linearised ADH plasmid for all template concentrations and assays (*p*<0.04) (Figure 4 & Appendix S1). This manifested as increased variability associated with the genomic template when compared to the linearised ADH plasmid (Figure 4). Bioanalyser and agarose gel analysis indicated that both the genomic and linearised ADH plasmid DNA were not highly fragmented (Figure S1) suggesting that the increased structural complexity of the gDNA may have led to the increased variability observed.

## Discussion

In this study we have evaluated how duplexing primer sets impact on dPCR analysis and, when template quantity is limiting, whether it is preferable or not to pre-amplify the template to increase the quantity. We have also used the ‘linked molecule’ design of the experiment to investigate the inherent dPCR technical variability, independent of upstream factors that alter the amount of template.

Previously it has been hypothesised that duplex dPCR could increase the accuracy in detection of CNVs over uniplex dPCR due to the reduced number of cumulative pipetting steps [27]. Our data demonstrated that while both the uniplex and duplex assay formats are fit for purpose for analysis of copy number ratios using both linearised plasmid and complex templates, the more precise measurement afforded by the duplex assays would allow a smaller difference in ratios between two samples to be differentiated. Furthermore, duplexing a PCR experiment has considerable advantages over uniplex reactions for a wide range of applications such as molecular diagnostics and forensic analysis [28]–[30]. Samples where low quantity template is commonplace, for example in cell free DNA analysis [31], [32], can benefit from duplexing as it reduces the number of separate PCR reactions required [25] and thus amount of sample used. Technical benefits also include reduction in cumulative errors incurred by pipetting and template heterogeneity, which would also be advantageous in the detection of CNVs where the difference in the number of molecules of two different targets is the key measurement [27].

Recent publications have established that dPCR has improved measurement precision over qPCR [2], [18], [33] and here we have demonstrated that dPCR precision can be improved further with the use of duplex reactions (Figure 1). A further parameter affecting the level of precision offered by dPCR is template concentration, where low template concentration (<200 positive chambers per panel that equates to *λ* <0.30) can produce less precision in the measurement than those that fall within the recommended range of accurate quantification (200 to 700 positive chambers per panel that equates to 0.30< *λ* <2.47) [2], [18], [24]. One possible solution for analysis of low concentration template is to perform a pre-amplification of the sample to increase amount of available template [34], [35]. However for this to be used for quantitative measurement, any bias introduced by the pre-amplification step needs to be negligible or clearly defined.

In an earlier study we observed a two-fold quantification bias when using a sequence-specific PCR-based pre-amplification approach [18]. As dPCR is capable of measuring DNA at low concentrations with good precision [2], [18] it was interesting to determine whether analysis of low concentration (*λ*) template, with the associated reduced sensitivity, was more accurate than performing pre-amplification to increase the template concentration and thus accuracy of dPCR, but at the risk of introducing bias associated with this additional step.

Our data have established that the precision afforded by dPCR at low *λ* can exceed that of the same template pre-amplified to increase the *λ* (Figure 3). The impact of such a loss of precision reduces the power of dPCR considerably as pre-amplification may not facilitate the resolution of fold changes below 1.5-fold that is currently possible using pre-amplification and gene expression analysis by qPCR [6]. Furthermore, our data also demonstrated that the pre-amplification bias observed was not consistent for a sample or assay between experiments and so cannot be compensated for in the analysis of the data set. This study also demonstrated the necessity of replicating the pre-amplification step as part of the experiment in question, thereby reducing the impact of the bias incurred in each pre-amplification reaction, but at the expense of loss of precision in this experiment.

The experiments performed here only looked at a single pre-amplification method that used a sequence-specific PCR based approach; it is possible that different approaches would afford better precision. However, other pre-amplification methods have also been shown to introduce a measurement bias [36], [37]. Regardless, of the method chosen, it is essential that the associated biases are defined. Identifying bias introduced during an experiment can be problematic and is commonly performed by normalising Cq values (ΔΔCq) [5], [7], [9], [17], [18]. The design of our study uses dPCR to calculate the relative quantity between two targets of interest as the metric for determining bias introduced by the pre-amplification step. As this method uses absolute quantification of each of the targets of interest, it could be more accurate at detecting the subtle bias that is undetectable using the normalising Cq method [18]. Therefore, our study provides a novel method for measuring the level of technical bias a pre-amplification step can introduce into a data set.

During analysis of the duplex dPCR experiments in this study, it was observed that some chambers exhibited amplification of only one assay. The design of the experiments is such that the different amplicons are present on the same molecule and therefore provides a method for specifically measuring the technical variation associated with molecular dropout. Our data identified the template type as having the largest technical variation with genomic DNA demonstrating a greater variability than linearised plasmid DNA. It was not clear why this difference occurred although the increased complexity of the gDNA may result in increased molecular dropout and thus variability, although potential causes could include the presence of inhibitors in the extracted gDNA sample, tertiary structures or nicks in the gDNA.

Other sources of error in dPCR that may also contribute to the variability in the measurement include chamber volume variation and sample distribution within a panel [38], degradation of the template due to prolonged periods of heating [39], [40], PCR inhibitors that affect one assay more than the other [3] as well as possible manual shearing of the DNA during microfluidic loading of the array. Overall, our findings suggest that care must be taken when linearised plasmid DNA is used to assess or control for more complex templates like genomic DNA. It is important that any difference in measurement performance are defined and ideally reduced to a minimum. This is directly relevant to molecular applications where surrogate molecules, like plasmids, are used to control and monitor assay performance or as reference materials for calibration [41], [42].

### Conclusions

In conclusion, the findings of this study address a number of issues associated with dPCR and provide guidance when performing fine quantitative molecular measurements using small amounts of material. We demonstrated that performing duplex dPCR can be more precise than uniplex and, when template is limiting, performing pre-amplification may not be necessary as it does not improve the measurement of the low concentration sample. We have also demonstrated with this study that pre-amplification bias is not systematic and so compensation in the analysis of the data set would be challenging. These findings are of importance as high throughput technologies, such as next generation sequencing or microfluidic qPCR, that rely on a pre-amplification reaction become more established to increase the amount of sample prior to quantification. We have also looked at molecular dropout as the source of dPCR technical variability and our data have shown that this can differ when different DNA templates are used. These findings further support the notion that dPCR is a highly accurate method for directly performing inter-assay comparisons, but that there are factors, including template type, that must be considered that can impact on technical performance.

## Supporting Information

Figure S1 **Assessment of template DNA for qPCR and dPCR analysis.** **A)** Analysis of approximately 16 ng *Bgl*I linearised ADH plasmid using the 2100 Bioanalyzer and DNA 7500 Series II kit according to the manufacturer’s instructions (Agilent). Electropherogram shows a single peak of ∼4.6 kb confirming complete linearisation of the ADH plasmid. The lower and upper markers are shown with green (50 bp) and purple (10380 bp) labelled peaks. **B)** Agarose gel analysis of *Arabidopsis* gDNA showing high molecular weight gDNA of ∼40 kb. gDNA dilutions (0.125 µg to 1 µg as indicated above each lane) were run with 1 X Gel Loading Dye (NEB) on a 1% agarose gel in 1 X TBE (SIGMA) and 1 X Gel Red (Biotimum). 300 ng of 1 kb extension DNA molecular ladder (Invitrogen) were run to size the gDNA.
(JPG)

Figure S2 **Adh assay information.** **A)** Schematic showing the three linked Adh assays on the *Arabidopsis thaliana landsberg* alcohol dehydrogenase (ADH) gene fragment (GenBank: M12196) that is cloned into the pSP64 poly(A) plasmid. The position within the NCBI database is given below the schematic. The distance from the centre of each Adh assay is given above the schematic. The schematic is not too scale. **B)** The amplification of a single PCR product for each Adh assay was confirmed using the 2100 Bioanalyzer and DNA 1000 kit (Agilent) according to the manufacturer’s instructions. Traces shown are amplified from the linearised ADH plasmid. **C–E)** SDS v2.4 software (ABI) generated amplification plots. For example Adhα-FAM assay in uniplex (C), Adhα-FAM assay in duplex (D) with Adhβ-VIC (not shown) and NTCs for all three Adh assays in both uniplex and duplex formats (E). **F–H)** Digital PCR analysis software (Fluidigm) generated heat maps and amplification plots. Amplification curves for each panel are show underneath their respective heat maps. Horizontal lines in the amplification plots represent the Cq threshold while the two vertical lines represent the Cq target range. For example Adhα-FAM assay (red) in uniplex (F), Adhα-FAM assay (red) in duplex with Adhβ-VIC (blue) (G) and NTC for Adhα-FAM:Adhβ-VIC duplex assay (H).
(JPG)

Figure S3 **Assessment of uniplex and duplex assays by real-time quantitative PCR.** Standard curves for each of the six Adh assays in uniplex (Adhα-FAM, Adhα-VIC, Adhβ-FAM, Adhβ-VIC, Adhδ-FAM and Adhδ-VIC) and for the six possible combinations of the Adh assays in duplex (Adhα-FAM:Adhβ-VIC, Adhβ-FAM:Adhα-VIC, Adhα:Adhδ-VIC, Adhδ-FAM:Adhα-VIC, Adhβ-FAM:Adhδ-VIC and Adhδ-FAM:Adhβ-VIC). Each standard curve is generated from the three qPCR data points for each standard curve dilution (log scale) generated on three separate days (x-axis) plotted against the Cq value (y-axis). The linear correlation (R^2^) and PCR efficiencies (E % = (10^(−1/slope)^−1)×100) were calculated from the standard curve. This data is summarised in Table S5.
(JPG)

Figure S4 **Screen of pre-amplification experiments to determine the optimum dilution factor for downstream dPCR analysis.** **A–B)** qPCR was used to determine the dilution factor for the preamplification reaction for dPCR analysis. The preamplification reaction was serially diluted in 1 X TE (pH 8.0) to establish the dilution factor that most closely resembled the ‘high’ concentration (red curve). Four dilutions were assessed: 1∶5 (green curve), 1∶125 (blue curve), 1∶250 (grey curve) and 1∶750 (purple curve). The ‘low’ concentration that was used as the template in the preamplification reaction is also included (yellow curve). For example, analysis of the linearised ADH plasmid with the Adhα-FAM:Adhβ-VIC duplex assay for Adhβ-VIC assay in triplicate qPCRs (A) and the preamplification NTC (black curve) and PCR NTC (aqua curve) show no amplification (B). **C–F)** Digital PCR analysis software generated heat maps and amplification plots. Amplification curves for each panel are show underneath their respective heat maps. Horizontal lines in the amplification plots represent the Cq threshold while the two vertical lines represent the Cq target range. For example, the Adhα-FAM:Adhβ-VIC duplex assay where Adhα-FAM (red amplification curves and positive chambers) and Adhβ-VIC (blue amplification curves and positive chambers) are shown for ‘high’ concentration linearised ADH plasmid (C), ‘low’ concentration linearised ADH plasmid (D), preamplified linearised ADH plasmid (E) and the NTC (F).
(JPG)

Table S1 Adh assay primer and probe sequences. Adapted from Sanders *et al.*, 2011. All primers were HPLC purified (Sigma, Dorset, UK), resuspended in dH2O and stored as 100 µM stocks at −20°C. All MGB probes were ordered HPLC purified at 100 µM concentration (ABI, California, USA) and stored at −20°C. All Adh assays had a final concentration 900 nM each of forward and reverse primers with 200 nM of the relevant MGB hydrolysis probe (conjugated with either a FAM or VIC fluorophore) in each PCR. Key: (F) forward primer, (R) reverse primer, (P1) FAM probe, (P2) VIC probe, MGB minor groove binder.
(DOCX)

Table S2 MIQE checklist. Minimum Information for publication of Quantitative real-time PCR Experiments checklist for authors, reviewers and editors.
(DOCX)

Table S3 Data for Figure 1 Absolute counts from 12.765 and 48.770 digital arrays. Ratio, 95% CI low and 95% CI high were calculated using the equations defined in Whale *et al.*, 2012. In all cases, the observed targets per panel were approximately half the expected number of targets per panel based on Nanodrop UV spectrophotometry using A260 measurement readings as reported previously (Sanders *et al.*, 2011).
(XLSX)

Table S4 Data for Figure 3 Absolute counts from 48.770 digital arrays. Ratio, 95% CI low and 95% CI high were calculated using the equations defined in Whale *et al.*, 2012. In all cases, the observed targets per panel were approximately half the expected number of targets per panel based on Nanodrop UV spectrophotometry using A260 measurement readings as reported previously (Sanders *et al.*, 2011).
(XLSX)

Table S5 Assessment of uniplex and duplex reactions by real-time PCR. Each standard curve was generated from a seven-point ten-fold dilution series of the linearised ADH plasmid (2×10^7^ to 2×10^1^ copies/reaction). Each dilution was analysed once by qPCR for each Adh assay in both uniplex and duplex and repeated on three separate days from a fresh standard curve to give three data points for each dilution and uniplex or duplex combination. The PCR efficiencies (E %) and linear correlations (R^2^) were calculated from this combined data set. E% was calculated using the formula (10^(−1/slope)^ −1)×100. The data summarised in this table is shown in Figure S3. Key: MGB, Minor Groove Binder; Ave, mean average of three experiments; Cq, quantification cycle; R^2^, linear correlation; T-test, two-tailed with equal variance.
(DOCX)

Appendix S1 Multinomial statistical model.
(DOCX)
