# Supplementary figures and images for: Methods for Applying Accurate Digital PCR Analysis on Low Copy DNA Samples
Source: PLoS One. 2013 Mar 5;8(3):e58177. doi: 10.1371/journal.pone.0058177 (PMC3589384; doi:10.1371/journal.pone.0058177)

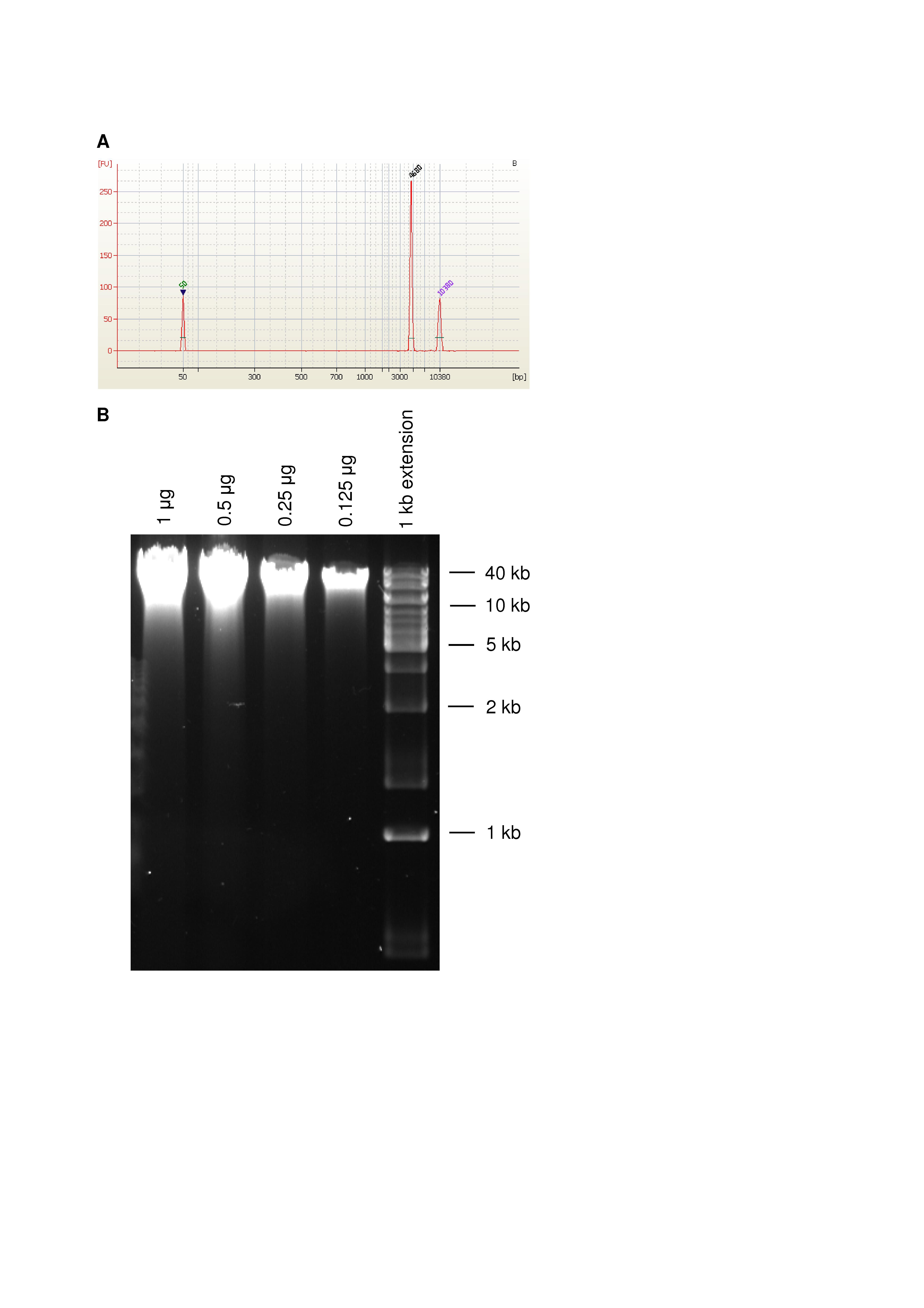

Supplement: Figure S1 — Assessment of template DNA for qPCR and dPCR analysis. A) Analysis of approximately 16 ng BglI linearised ADH plasmid using the 2100 Bioanalyzer and DNA 7500 Series II kit according to the manufacturer’s instructions (Agilent). Electropherogram shows a single peak of ∼4.6 kb confirming complete linearisation of the ADH plasmid. The lower and upper markers are shown with green (50 bp) and purple (10380 bp) labelled peaks. B) Agarose gel analysis of Arabidopsis gDNA showing high molecular weight gDNA of ∼40 kb. gDNA dilutions (0.125 µg to 1 µg as indicated above each lane) were run with 1 X Gel Loading Dye (NEB) on a 1% agarose gel in 1 X TBE (SIGMA) and 1 X Gel Red (Biotimum). 300 ng of 1 kb extension DNA molecular ladder (Invitrogen) were run to size the gDNA. (JPG) [file pone.0058177.s001.jpg]

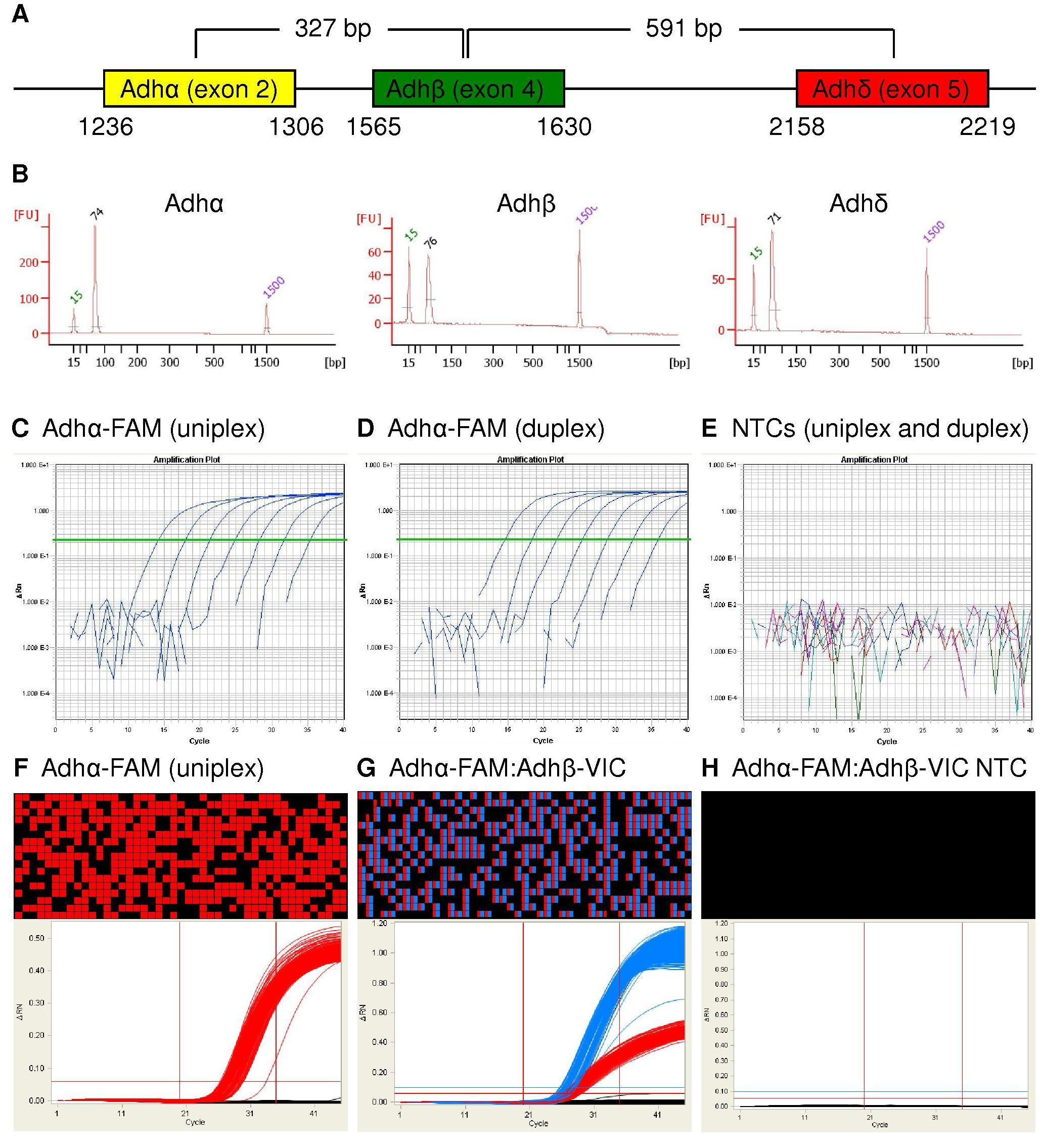

Supplement: Figure S2 — Adh assay information. A) Schematic showing the three linked Adh assays on the Arabidopsis thaliana landsberg alcohol dehydrogenase (ADH) gene fragment (GenBank: M12196) that is cloned into the pSP64 poly(A) plasmid. The position within the NCBI database is given below the schematic. The distance from the centre of each Adh assay is given above the schematic. The schematic is not too scale. B) The amplification of a single PCR product for each Adh assay was confirmed using the 2100 Bioanalyzer and DNA 1000 kit (Agilent) according to the manufacturer’s instructions. Traces shown are amplified from the linearised ADH plasmid. C–E) SDS v2.4 software (ABI) generated amplification plots. For example Adhα-FAM assay in uniplex (C), Adhα-FAM assay in duplex (D) with Adhβ-VIC (not shown) and NTCs for all three Adh assays in both uniplex and duplex formats (E). F–H) Digital PCR analysis software (Fluidigm) generated heat maps and amplification plots. Amplification curves for each panel are show underneath their respective heat maps. Horizontal lines in the amplification plots represent the Cq threshold while the two vertical lines represent the Cq target range. For example Adhα-FAM assay (red) in uniplex (F), Adhα-FAM assay (red) in duplex with Adhβ-VIC (blue) (G) and NTC for Adhα-FAM:Adhβ-VIC duplex assay (H). (JPG) [file pone.0058177.s002.jpg]

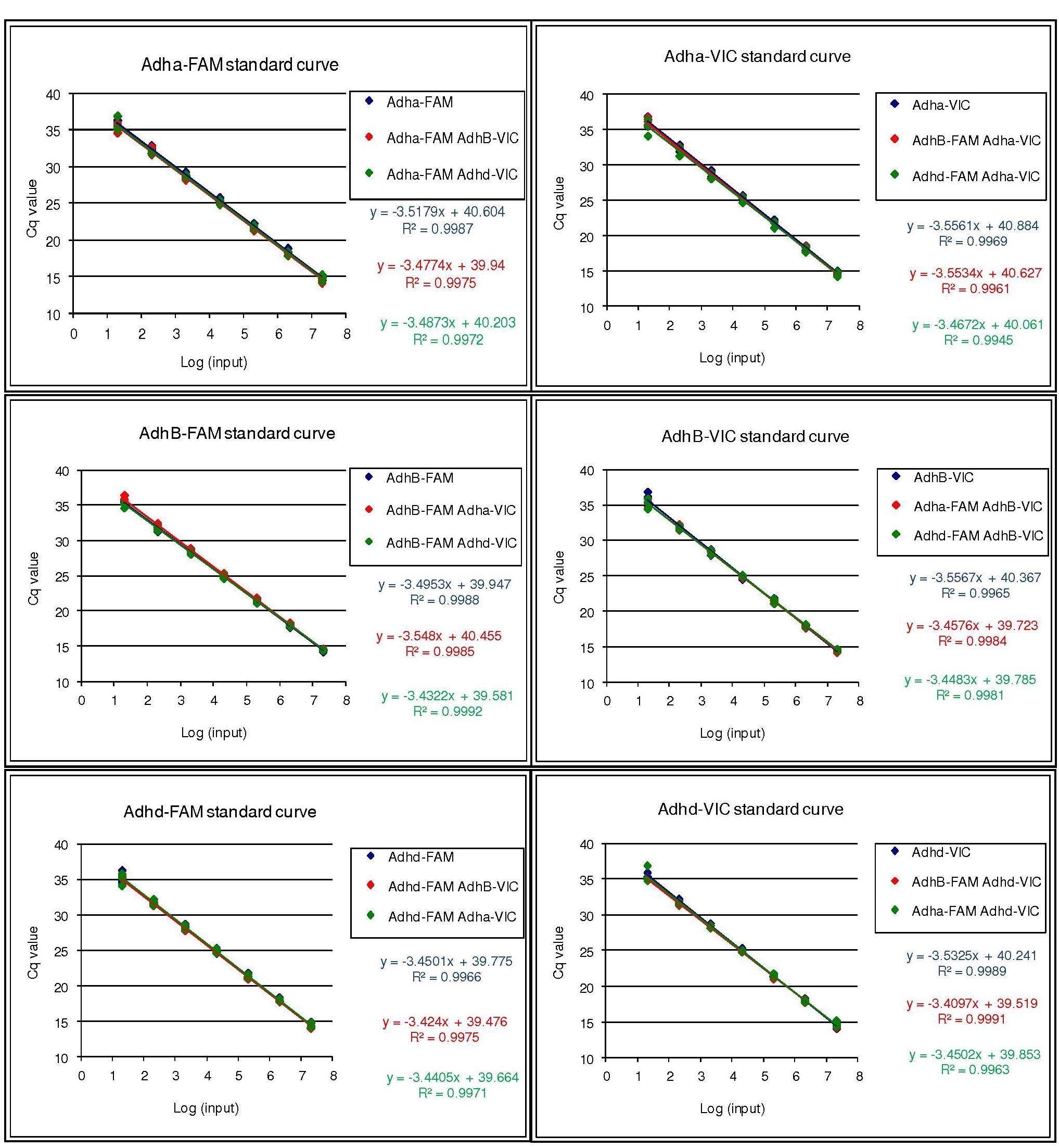

Supplement: Figure S3 — Assessment of uniplex and duplex assays by real-time quantitative PCR. Standard curves for each of the six Adh assays in uniplex (Adhα-FAM, Adhα-VIC, Adhβ-FAM, Adhβ-VIC, Adhδ-FAM and Adhδ-VIC) and for the six possible combinations of the Adh assays in duplex (Adhα-FAM:Adhβ-VIC, Adhβ-FAM:Adhα-VIC, Adhα:Adhδ-VIC, Adhδ-FAM:Adhα-VIC, Adhβ-FAM:Adhδ-VIC and Adhδ-FAM:Adhβ-VIC). Each standard curve is generated from the three qPCR data points for each standard curve dilution (log scale) generated on three separate days (x-axis) plotted against the Cq value (y-axis). The linear correlation (R2) and PCR efficiencies (E % = (10(−1/slope)−1)×100) were calculated from the standard curve. This data is summarised in Table S5. (JPG) [file pone.0058177.s003.jpg]

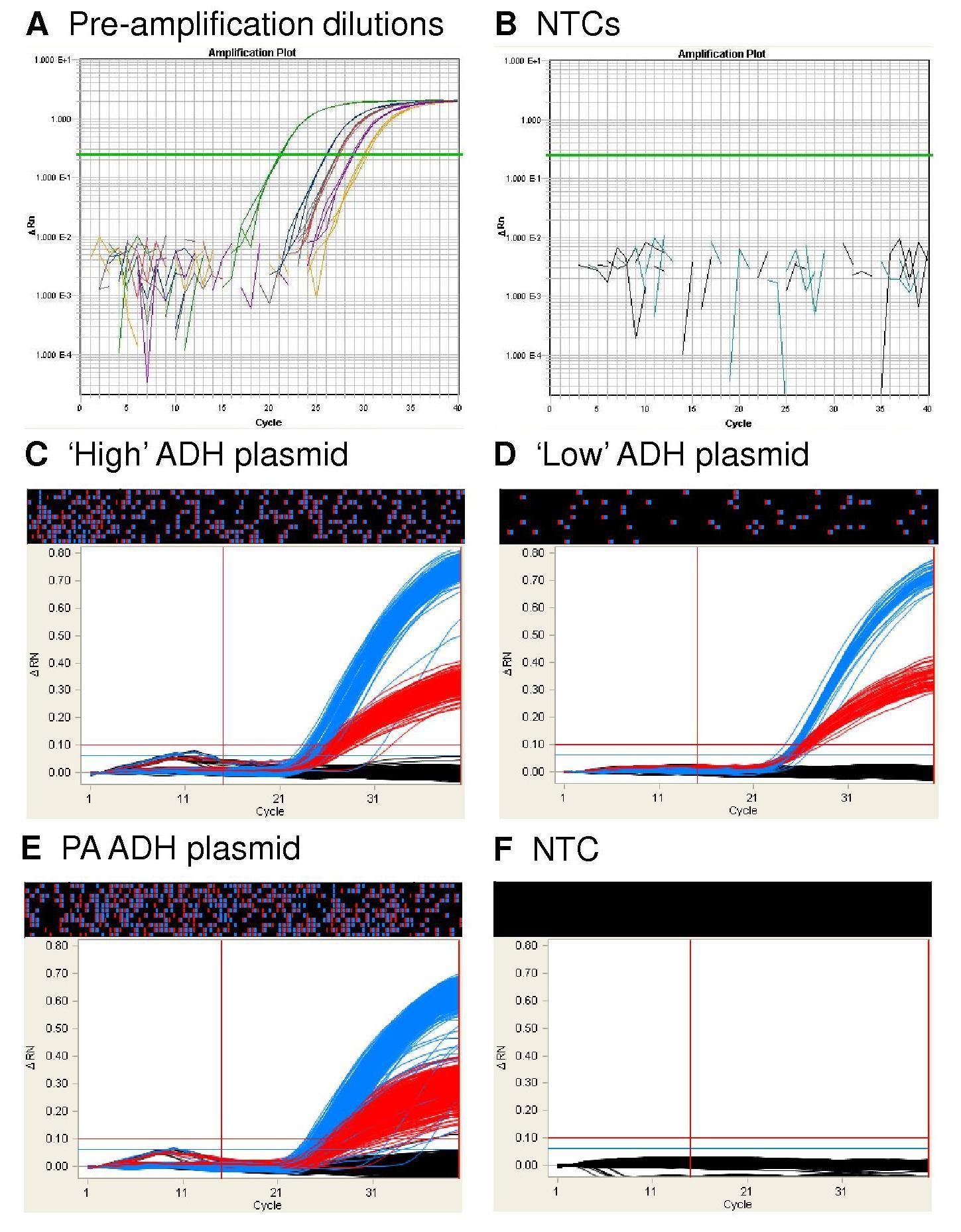

Supplement: Figure S4 — Screen of pre-amplification experiments to determine the optimum dilution factor for downstream dPCR analysis. A–B) qPCR was used to determine the dilution factor for the preamplification reaction for dPCR analysis. The preamplification reaction was serially diluted in 1 X TE (pH 8.0) to establish the dilution factor that most closely resembled the ‘high’ concentration (red curve). Four dilutions were assessed: 1∶5 (green curve), 1∶125 (blue curve), 1∶250 (grey curve) and 1∶750 (purple curve). The ‘low’ concentration that was used as the template in the preamplification reaction is also included (yellow curve). For example, analysis of the linearised ADH plasmid with the Adhα-FAM:Adhβ-VIC duplex assay for Adhβ-VIC assay in triplicate qPCRs (A) and the preamplification NTC (black curve) and PCR NTC (aqua curve) show no amplification (B). C–F) Digital PCR analysis software generated heat maps and amplification plots. Amplification curves for each panel are show underneath their respective heat maps. Horizontal lines in the amplification plots represent the Cq threshold while the two vertical lines represent the Cq target range. For example, the Adhα-FAM:Adhβ-VIC duplex assay where Adhα-FAM (red amplification curves and positive chambers) and Adhβ-VIC (blue amplification curves and positive chambers) are shown for ‘high’ concentration linearised ADH plasmid (C), ‘low’ concentration linearised ADH plasmid (D), preamplified linearised ADH plasmid (E) and the NTC (F). (JPG) [file pone.0058177.s004.jpg]
