# Supplementary material for: Methods for Applying Accurate Digital PCR Analysis on Low Copy DNA Samples
Source: PLoS One. 2013 Mar 5;8(3):e58177. doi: 10.1371/journal.pone.0058177 (PMC3589384; doi:10.1371/journal.pone.0058177)
Supplement: Table S1 — Adh assay primer and probe sequences. Adapted from Sanders et al., 2011. All primers were HPLC purified (Sigma, Dorset, UK), resuspended in dH2O and stored as 100 µM stocks at −20°C. All MGB probes were ordered HPLC purified at 100 µM concentration (ABI, California, USA) and stored at −20°C. All Adh assays had a final concentration 900 nM each of forward and reverse primers with 200 nM of the relevant MGB hydrolysis probe (conjugated with either a FAM or VIC fluorophore) in each PCR. Key: (F) forward primer, (R) reverse primer, (P1) FAM probe, (P2) VIC probe, MGB minor groove binder. (DOCX) [file pone.0058177.s005.docx]

| **Table S1. Adh assay primer and probe sequences.** | |  |  |  |
| --- | --- | --- | --- | --- |
|  |  |  |  |  |
| **Target DNA (gDNA location)** | **Primer/Probe Sequence (5’ to 3’)** | **Amplicon size (bp)** | **Region of M12196** | **[reaction]** |
| Adhα assay (exon 2) | (F) GGTTGCTCCACCGCAGAA | 71 | 1236 – 2306 | 900 nM |
|  | (R) AAACATCGGTGTGACAGAGAGAAG |  |  | 900 nM |
|  | (P1) FAM-AAGTTCGTATCAAGATTC-MGB |  |  | 200 nM |
|  | (P2) VIC-AAGTTCGTATCAAGATTC-MGB |  |  |  |
| Adhβ assay (exon 4) | (F) TTGAGAGTGTTGGAGAAGGAGTGA | 66 | 1565 – 1630 | 900 nM |
|  | (R) CGGTAAAGATCGGCAACACA |  |  | 900 nM |
|  | (P1) FAM-TCTTCAGCCAGGAGATC-MGB |  |  | 200 nM |
|  | (P2) VIC-TCTTCAGCCAGGAGATC-MGB |  |  |  |
| Adhδ assay (exon 5) | (F) TGAACCCGAAAGACCATGACA | 62 | 2158 – 2219 | 900 nM |
|  | (R) CCCACCATCCGTCATCTCA |  |  | 900 nM |
|  | (P1) FAM-CCAATTCAACAGGTGATC-MGB |  |  | 200 nM |
|  | (P2) VIC-CCAATTCAACAGGTGATC-MGB |  |  |  |
|  | | | | |
|  |  |  |  |  |
|  |  |  |  |  |
|  |  |  |  |  |
