# Supplementary material for: Methods for Applying Accurate Digital PCR Analysis on Low Copy DNA Samples
Source: PLoS One. 2013 Mar 5;8(3):e58177. doi: 10.1371/journal.pone.0058177 (PMC3589384; doi:10.1371/journal.pone.0058177)
Supplement: Table S2 — MIQE checklist. Minimum Information for publication of Quantitative real-time PCR Experiments checklist for authors, reviewers and editors. (DOCX) [file pone.0058177.s006.docx]

| **Table S2: MIQE checklist.** |  |  |  |
| --- | --- | --- | --- |
|  |  |  |  |
| **ITEM TO CHECK** | **IMPORTANCE** | **CHECKLIST** | **COMMENTS/WHERE?** |
| **EXPERIMENTAL DESIGN** |  |  |  |
| Definition of experimental and control groups | **E** | **YES** | Materials and Methods |
| Number within each group | **E** | **YES** | Materials and Methods |
| Assay carried out by core lab or investigator's lab? | D | **YES** | Investigator's lab |
| Acknowledgement of authors' contributions | D | **YES** | Author Information |
| **SAMPLE** |  |  |  |
| Description | **E** | **N/A** |  |
| Volume/mass of sample processed | D | **N/A** |  |
| Microdissection or macrodissection | **E** | **N/A** |  |
| Processing procedure | **E** | **N/A** |  |
| If frozen - how and how quickly? | **E** | **N/A** |  |
| If fixed - with what, how quickly? | **E** | **N/A** |  |
| Sample storage conditions and duration (especially for FFPE samples) | **E** | **N/A** |  |
| **NUCLEIC ACID EXTRACTION** |  |  |  |
| Procedure and/or instrumentation | **E** | **YES** | Materials and Methods |
| Name of kit and details of any modifications | **E** | **N/A** |  |
| Source of additional reagents used | D | **N/A** |  |
| Details of DNase or RNAse treatment | **E** | **N/A** |  |
| Contamination assessment (DNA or RNA) | **E** | **YES** | Materials and Methods |
| Nucleic acid quantification | **E** | **YES** | Materials and Methods |
| Instrument and method | **E** | **YES** | Materials and Methods, Supplementary Figure 1 |
| Purity (A260/A280) | D | **YES** | Nanodrop (Thermal Scientific) |
| Yield | D | **YES** | Materials and Methods |
| RNA integrity method/instrument | **E** | **N/A** |  |
| RIN/RQI or Cq of 3' and 5' transcripts | **E** | **N/A** |  |
| Electrophoresis traces | D | **N/A** |  |
| Inhibition testing (Cq dilutions, spike or other) | **E** | **N/A** |  |
| **REVERSE TRANSCRIPTION** |  |  |  |
| Complete reaction conditions | **E** | **N/A** |  |
| Amount of RNA and reaction volume | **E** | **N/A** |  |
| Priming oligonucleotide (if using GSP) and concentration | **E** | **N/A** |  |
| Reverse transcriptase and concentration | **E** | **N/A** |  |
| Temperature and time | **E** | **N/A** |  |
| Manufacturer of reagents and catalogue numbers | D | **N/A** |  |
| Cqs with and without RT | D* | **N/A** |  |
| Storage conditions of cDNA | D | **N/A** |  |
| **qPCR TARGET INFORMATION** |  |  |  |
| If multiplex, efficiency and LOD of each assay. | **E** | **YES** | Supplementary Figure 3 |
| Sequence accession number | **E** | **YES** | Materials and Methods and Supplementary Table 1 |
| Location of amplicon | D | **YES** | Supplementary Table 1 |
| Amplicon length | **E** | **YES** | Supplementary Table 1 |
| *In silico* specificity screen (BLAST, etc) | **E** | **YES** | Performed previously in Sanders *et al.*, 2011 |
| Pseudogenes, retropseudogenes or other homologs? | D | **YES** | Performed previously in Sanders *et al.*, 2011 |
| Sequence alignment | D | **YES** | Performed previously in Sanders *et al.*, 2011 |
| Secondary structure analysis of amplicon | D | **YES** | Performed previously in Sanders *et al.*, 2011 |
| Location of each primer by exon or intron (if applicable) | **E** | **YES** | Supplementary Table 1 |
| What splice variants are targeted? | **E** | **YES** | *Arabidopsis thaliana landsberg* variant |
| **qPCR OLIGONUCLEOTIDES** |  |  |  |
| Primer sequences | **E** | **YES** | Supplementary Table 1 |
| RTPrimerDB Identification Number | D | **N/A** |  |
| Probe sequences | D** | **YES** | Supplementary Table 1 |
| Location and identity of any modifications | **E** | **YES** | Supplementary Table 1 |
| Manufacturer of oligonucleotides | D | **YES** | Primers (SIGMA) and probes (ABI) |
| Purification method | D | **YES** | HPLC |
| **qPCR PROTOCOL** |  |  |  |
| Complete reaction conditions | **E** | **YES** | Materials and Methods |
| Reaction volume and amount of cDNA/DNA | **E** | **YES** | Materials and Methods |
| Primer, (probe), Mg++ and dNTP concentrations | **E** | **YES** | Materials and Methods, Manufactures proprietory |
| Polymerase identity and concentration | **E** | **YES** | AmpliTaq Gold® DNA Polymerase, UP (Ultra Pure) |
| Buffer/kit identity and manufacturer | **E** | **YES** | TaqMan® Gene Expression Mastermix (PN 4369016) |
| Exact chemical constitution of the buffer | D | **NO** | Manufactures proprietory |
| Additives (SYBR Green I, DMSO, etc.) | **E** | **N/A** | Hydrolysis probe quantification only |
| Manufacturer of plates/tubes and catalog number | D | **YES** | ABI 96-well plates (PN 4306737) |
| Complete thermocycling parameters | **E** | **YES** | Materials and Methods |
| Reaction setup (manual/robotic) | D | **YES** | Manual setup |
| Manufacturer of qPCR instrument | **E** | **YES** | Materials and Methods (ABI and Fluidigm) |
| **qPCR VALIDATION** |  |  |  |
| Evidence of optimisation (from gradients) | D | **YES** | Supplementary Figure 3 |
| Specificity (gel, sequence, melt, or digest) | **E** | **NO** | Supplementary Figure 2 |
| For SYBR Green I, Cq of the NTC | **E** | **N/A** | No SYBR Green assays used |
| Standard curves with slope and y-intercept | **E** | **YES** | Supplementary Figures 2 & 3 |
| PCR efficiency calculated from slope | **E** | **YES** | Supplementary Figure 3 |
| Confidence interval for PCR efficiency or standard error | D | **N/A** |  |
| r2 of standard curve | **E** | **YES** | Supplementary Figure 3 |
| Linear dynamic range | **E** | **YES** | Supplementary Figure 3 |
| Cq variation at lower limit | **E** | **YES** | Supplementary Figure 3 |
| Confidence intervals throughout range | D | **YES** | Supplementary Figure 3 |
| Evidence for limit of detection | **E** | **YES** | Supplementary Figure 3 |
| If multiplex, efficiency and LOD of each assay. | **E** | **YES** | Supplementary Figure 3 |
| **DATA ANALYSIS** |  |  |  |
| qPCR analysis program (source, version) | **E** | **YES** | Materials and Methods |
| Cq method determination | **E** | **YES** | Materials and Methods |
| Outlier identification and disposition | **E** | **YES** | No outliers identified |
| Results of NTCs | **E** | **YES** | Materials and Methods, Supplementary Figure 2 |
| Justification of number and choice of reference genes | **E** | **N/A** | Standard curve quantification |
| Description of normalisation method | **E** | **YES** | Standard curve quantification |
| Number and concordance of biological replicates | D | **N/A** |  |
| Number and stage (RT or qPCR) of technical replicates | **E** | **YES** | Materials and Methods |
| Repeatability (intra-assay variation) | **E** | **YES** | Results |
| Reproducibility (inter-assay variation, %CV) | D | **YES** | Results |
| Power analysis | D | **No** | Not applicable |
| Statistical methods for result significance | **E** | **YES** | Materials and Methods/Supplemental |
| Software (source, version) | **E** | **YES** | Materials and Methods/ Supplemental |
| Cq or raw data submission using RDML | D | **N/A** |  |
|  | | | |
| All essential information (E) must be submitted with the manuscript. Desirable information (D) should be submitted if available. If using primers obtained from RTPrimerDB, information on qPCR target, oligonucleotides, protocols and validation is available from that source. | | | |
|  | | | |
| *: Assessing the absence of DNA using a no RT assay is essential when first extracting RNA. Once the sample has been validated as DNA-free, inclusion of a no-RT control is desirable, but no longer essential. | | | |
|  |  |  |  |
|  |  |  |  |
| **: Disclosure of the probe sequence is highly desirable and strongly encouraged. However, since not all commercial pre-designed assay vendors provide this information, it cannot be an essential requirement. Use of such assays is advised against. | | | |
|  |  |  |  |
