# Supplementary material for: Methods for Applying Accurate Digital PCR Analysis on Low Copy DNA Samples
Source: PLoS One. 2013 Mar 5;8(3):e58177. doi: 10.1371/journal.pone.0058177 (PMC3589384; doi:10.1371/journal.pone.0058177)
Supplement: Table S5 — Assessment of uniplex and duplex reactions by real-time PCR. Each standard curve was generated from a seven-point ten-fold dilution series of the linearised ADH plasmid (2×10^7^ to 2×101 copies/reaction). Each dilution was analysed once by qPCR for each Adh assay in both uniplex and duplex and repeated on three separate days from a fresh standard curve to give three data points for each dilution and uniplex or duplex combination. The PCR efficiencies (E %) and linear correlations (R2) were calculated from this combined data set. E% was calculated using the formula (10(−1/slope) −1)×100. The data summarised in this table is shown in Figure S3. Key: MGB, Minor Groove Binder; Ave, mean average of three experiments; Cq, quantification cycle; R2, linear correlation; T-test, two-tailed with equal variance. (DOCX) [file pone.0058177.s009.docx]

**Table S5. Assessment of uniplex and duplex reactions by real-time PCR.**

| Type of assay | MGB probe type | | FAM-MGB | | | VIC-MGB | | | Comparison of uniplex versus duplex (Student’s t-test *p* value) | | | |
| --- | --- | --- | --- | --- | --- | --- | --- | --- | --- | --- | --- | --- |
|  | FAM | VIC | Ave Cq | E% | R^2^ | Ave Cq | E% | R^2^ | FAM | | VIC | |
|  |  |  |  |  |  |  |  |  | Ave Cq | E% | Ave Cq | E% |
| Uniplex | Adhα |  | 25.47 | 92.42 | 0.9987 |  |  |  | 0.43 | 0.54 | 0.24 | 0.02 |
|  | Adhβ |  | 24.91 | 93.24 | 0.9988 |  |  |  |  | |  | |
|  | Adhδ |  | 24.94 | 94.92 | 0.9966 |  |  |  |  |  |  |  |
|  |  | Adhα |  |  |  | 25.59 | 91.08 | 0.9969 |  |  |  |  |
|  |  | Adhβ |  |  |  | 25.07 | 91.06 | 0.9965 |  |  |  |  |
|  |  | Adhδ |  |  |  | 25.05 | 91.90 | 0.9989 |  |  |  |  |
| Duplex | Adhα | Adhβ | 24.98 | 93.90 | 0.9975 | 24.85 | 94.63 | 0.9984 |  |  |  |  |
|  | Adhβ | Adhα | 25.20 | 95.60 | 0.9992 | 25.34 | 91.17 | 0.9961 |  |  |  |  |
|  | Adhα | Adhδ | 25.20 | 93.53 | 0.9972 | 25.01 | 94.91 | 0.9963 |  |  |  |  |
|  | Adhδ | Adhα | 24.87 | 95.28 | 0.9971 | 25.15 | 94.28 | 0.9945 |  |  |  |  |
|  | Adhβ | Adhδ | 24.82 | 91.36 | 0.9985 | 24.85 | 96.46 | 0.9991 |  |  |  |  |
|  | Adhδ | Adhβ | 24.75 | 95.91 | 0.9975 | 24.95 | 94.98 | 0.9981 |  |  |  |  |
