# Supplementary material for: Methods for Applying Accurate Digital PCR Analysis on Low Copy DNA Samples
Source: PLoS One. 2013 Mar 5;8(3):e58177. doi: 10.1371/journal.pone.0058177 (PMC3589384; doi:10.1371/journal.pone.0058177)
Supplement: Appendix S1 — Multinomial statistical model. (DOCX) [file pone.0058177.s010.docx]

**Appendix S1**

**Multinomial Statistical Model**

The *multinom* function in the *nnet* R package was used to fit a multinomial log-linear model to the duplex count data. The proportions of the total count (equal to the panel size of 770) attributed to each detection outcome are *p*_0_, *p*_F_, *p*_V_ and *p*_FV_ where

$p_{0}+p_{\text{F}}+p_{\text{V}}+p_{\text{FV}}=1$ (1)

where 0, F, V and FV refer to the detection of neither assay, FAM only, VIC only, and both FAM and VIC. Expected (fitted) counts are obtained by multiplying these proportions by 770. The fitted model coefficients are in the form of log odds using the 0 category as a reference. Thus, for example, the log odds *L*_V_ for VIC only are equal to $\ln\left(p_{\text{V}}/p_{0}\right)$. The model is a linear model, and so log odds terms are estimated for each of the variables in the model and added together to get the overall log odds for a particular combination of values of the variables. The constraint of Equation 1 means that only three sets of terms are required to characterise the model completely. The reference proportion is given by

$p_{0}=\frac{1}{1+\exp L_{\text{F}}+\exp L_{\text{V}}+\exp L_{\text{FV}}}$ (2)

which allows the other three proportions to be computed from their respective log odds.

**α-FAM/β-VIC**

The minimal model which adequately explains the observed counts includes Experiment, Template and Concentration, plus an interaction between Experiment and Template. Fitted log odds terms are given in the table below, with standard errors in brackets, and show that the strongest contributors are Template and Concentration. Experimental variation is essentially a random effect, but we were not able to model it as such; consequently Experiment is used only to extract the concentration and template effects.

To illustrate how the table is used, consider the VIC-only count in the low-concentration plasmid group in experiment 3. The reference group is high-concentration genomic DNA in experiment 1, and has log odds equal to −4.12. To compute the log odds for our group we take the reference and add the contributions for the differences as follows:

$$L_{\text{V}}=-4.11+0.06-1.66-1.91-0.57=-8.19$$

For experiment 3 we add 0.06, for plasmid instead of gDNA we subtract 1.66, and for low concentration instead of high we subtract 1.90. Because there is an interaction between Experiment and Template, one further subtraction is necessary, to represent Expt 3 with plasmid DNA, giving a final log odds figure of −8.19. If required, the standard error in *L*_V_ can be computed, using the variance-covariance matrix produced by inverting the Hessian matrix (not shown here).

For our group, the expected number of VIC-only chambers is lower than for the reference group, indicating that the number of chambers subject to dropout of FAM is lower. This is partly due to experimental variability, as well as the lower concentration leading to lower counts generally, but is also because plasmid DNA results in less dropout than does genomic DNA.

|  | FAM only | VIC only | Both FAM and VIC |
| --- | --- | --- | --- |
| *Intercept (reference group)*  Expt 1, gDNA, High Conc | −4.26 (0.19) | −4.12 (0.17) | −0.522 (0.035) |
| Expt 3 | 0.04 (0.26) | 0.06 (0.23) | 0.027 (0.048) |
| Expt 5 | −0.24 (0.27) | −0.02 (0.24) | −0.160 (0.049) |
| Plasmid | −1.76 (0.45) | −1.66 (0.39) | −0.332 (0.049) |
| Low Conc | −2.23 (0.32) | −1.90 (0.25) | −1.940 (0.036) |
| Expt 3/Plasmid | −0.48 (0.70) | −0.57 (0.62) | −0.159 (0.070) |
| Expt 5/Plasmid | 0.04 (0.66) | −0.12 (0.57) | 0.109 (0.070) |

The statistical significance of the different terms was assessed by progressive model reduction and comparison of Akaike Information Criterion (AIC) values. Where appropriate, likelihood ratio tests on the residual deviance of different models were also carried out. Removal of the interaction term produced an increase in deviance which was significant at the 95% confidence level (*p* = 0.014); it was accordingly retained in the model.

**δ-FAM/β-VIC and β-FAM/ δ-VIC**

The minimal model contains Experiment, Template and Concentration, with an interaction between Template and Concentration. Fitted model terms are given in the table below. Again, Concentration and Template are the strongest contributors. The combination of assay with fluorophore was not found to be significant.

The interaction term was tested for significance with a likelihood ratio test on the model deviance and found to be borderline significant at the 95% level (*p* = 0.033).

|  | FAM only | VIC only | Both FAM and VIC |
| --- | --- | --- | --- |
| *Intercept (reference group)*  Expt 2, gDNA, High Conc | −3.731 (0.094) | −3.90 (0.10) | −0.563 (0.021) |
| Expt 4 | −0.02 (0.12) | −0.04 (0.13) | −0.177 (0.024) |
| Expt 6 | −0.07 (0.13) | 0.08 (0.13) | 0.046 (0.023) |
| Plasmid | −1.59 (0.15) | −1.49 (0.15) | 0.210 (0.022) |
| Low Conc | −1.72 (0.15) | −1.55 (0.15) | −1.972 (0.037) |
| Plasmid/Low Conc | −0.26 (0.36) | −1.21 (0.49) | −0.037 (0.048) |

Using the tables of fitted coefficients and Equations 1 and 2, the expected counts in each category can be calculated. The tables below give approximate estimates which have been averaged over the three experiments. Confidence intervals on these figures are not given, but this can be done in principle

α-FAM/β-VIC:

| Template | Concentration | Neither | FAM only | VIC only | Both |
| --- | --- | --- | --- | --- | --- |
| gDNA | High | 481.9 | 6.4 | 7.9 | 273.7 |
| gDNA | Low | 709.2 | 1.0 | 1.7 | 58.0 |
| Plasmid | High | 548.0 | 1.1 | 1.4 | 219.5 |
| Plasmid | Low | 727.7 | 0.2 | 0.3 | 41.9 |

δ-FAM/β-VIC and β-FAM/ δ-VIC:

| Template | Concentration | Neither | FAM only | VIC only | Both |
| --- | --- | --- | --- | --- | --- |
| gDNA | High | 484.4 | 11.3 | 10.0 | 264.4 |
| gDNA | Low | 709.9 | 2.9 | 3.1 | 54.1 |
| Plasmid | High | 457.8 | 2.2 | 2.1 | 308.0 |
| Plasmid | Low | 705.5 | 0.5 | 0.2 | 63.8 |

The tables show clearly that a low concentration results in a larger number of wells showing no response, and fewer wells for which either or both assays show a positive signal; this is because fewer wells contain any template, and does not relate to the probability of dropout. Plasmid DNA shows reduced counts compared to the equivalent concentration with genomic DNA when only one assay has been detected, and suggests that dropout is more prevalent with genomic DNA.

It is the counts relating to the detection of only one assay which give the most direct information about dropout, but we should note that the proportions estimated for the F and V classes are not in themselves dropout probabilities. Further work would be required to establish whether the probability of dropout depends on any of the factors explored in this work.
